# CAV1 promotes epithelial-to-mesenchymal transition (EMT) and chronic renal allograft interstitial fibrosis by activating the ferroptosis pathway

**DOI:** 10.3389/fimmu.2025.1523855

**Published:** 2025-02-12

**Authors:** Qianguang Han, Bin Ni, Wei Bao, Junqi Zhang, Ming Zheng, Jinxu Miu, Zijie Wang, Jingwen Yuan, Jun Tao, Zhijian Han, Min Gu, Xiaobing Ju, Ruoyun Tan

**Affiliations:** ^1^ Department of Urology, the First Affiliated Hospital of Nanjing Medical University, Nanjing, China; ^2^ Department of Urology, the Second Affiliated Hospital of Nanjing Medical University, Nanjing, China

**Keywords:** chronic renal graft dysfunction (CAD), epithelial-mesenchymal transition (EMT), ferroptosis, Caveolin-1(CAV1), Interleukin-6 (IL-6)

## Abstract

**Background:**

Chronic allograft dysfunction (CAD) stands as a critical factor that limits the long-term viability of transplanted kidneys. Ferroptosis is an iron-dependent form of programmed cell death increasingly linked to chronic fibrosis. However, the mechanism by which ferroptosis contributes to the onset and progression of CAD remains unclear.

**Methods:**

This study analyzed transcriptome data from renal transplant biopsy samples in the Gene Expression Omnibus (GEO), through clinical samples, animal models, and cell experiments, this study investigated the mechanism by which Caveolin-1 (CAV1) promotes CAD through the regulation of the ferroptosis pathway.

**Results:**

The elevated levels of CAV1 were found to positively correlate with CAD incidence. Clinical and animal model validation confirmed heightened CAV1 expression in CAD. *In vitro* experiments demonstrated that CAV1 can directly promote chronic renal allograft interstitial fibrosis by regulating ferroptosis in renal tubular epithelial cells; additionally, it can promote epithelial-to-mesenchymal transition (EMT) by secreting Interleukin- 6 (IL-6), thereby further contributing to CAD.

**Conclusion:**

CAV1 plays a critical role in the development of CAD by promoting EMT and chronic renal allograft interstitial fibrosis through the ferroptosis pathway. Adjusting ferroptosis by altering the expression abundance of CAV1 may become an important method for the prevention and treatment of CAD in the future.

## 1 Introduction

Kidney transplantation represents the optimal therapeutic approach for end-stage renal disease, markedly enhancing patient quality of life and substantially alleviating the burden of long-term hemodialysis (1). Post-renal transplantation rejection encompasses three primary forms of allograft rejection: hyperacute rejection, acute rejection (AR) and chronic rejection (2). Chronic rejection in renal transplantation clinically manifests as a slow deterioration in allograft function and represents the primary cause of delayed renal graft failure. Chronic transplant renal dysfunction (CAD) stems from a range of factors, including glomerulosclerosis, inflammation, interstitial fibrosis, and tubular atrophy (IF/TA) (3, 4). Epithelial-mesenchymal transition (EMT), serving as the primary source of interstitial matrix deposition, plays a crucial role in driving the development of CAD (5). The prevention or delay of CAD occurrence becomes a crucial long-term treatment goal following kidney transplantation.

Ferroptosis is a novel form of programmed cell death dependent on iron, distinguished from apoptosis, necrosis, and autophagy at the cellular level (6). The primary mechanism of ferroptosis involves the catalysis of heightened expression of unsaturated fatty acids on the cell membrane in the presence of divalent iron or esteroxygenase, leading to lipid peroxidation and subsequent cell death. Furthermore, ferroptosis is characterized by a reduction in the activity of the regulatory core enzyme GPX4 within the antioxidant system, specifically the glutathione system (7). Regarding cell morphology, ferroptosis induces smaller mitochondria, heightened membrane density, and diminished cristae. However, morphological alterations are not observed in the nucleus. Concerning cellular composition, ferroptosis is characterized by elevated lipid peroxidation and distinct genetic alterations. Utilizing iron agents and experiencing an overload of iron ions can lead to an accumulation of excessive iron in the cytoplasm and mitochondria, thereby hastening the production of highly toxic reactive oxygen species, particularly hydroxyl radicals, resulting in ferroptosis (8), With the occurrence of ferroptosis in the transplanted kidney tissues, it exacerbates the inflammatory response and secretes a large amount of validating and pro-fibrotic factors, which together promote the process of EMT, which in turn accelerates interstitial fibrosis and CAD in the transplanted kidney (9).

In recent years, the widespread adoption of genetic testing technologies has led to a gradual shift in research focus from macroscopic phenotypic studies to microscopic investigations of genes, signal transduction, and other cellular processes, especially with the development and application of second-generation sequencing and single-cell technologies. These advancements have further deepened the study of gene expression and the role of different cell subtypes (10, 11). In this study, datasets pertaining to chronic rejection after renal transplantation were retrieved from the GEO database. After screening, biopsy samples from the GSE21374 dataset were selected for analysis. Subsequently, the samples from GSE21374 were categorized into CAD and No-CAD groups based on the biopsy results (12). Differentially expressed genes were initially identified, followed by the extraction of differentially expressed ferroptosis-related genes through their association with known ferroptosis-related genes. Subsequently, prediction models were constructed utilizing these genes to identify target genes. Survival analysis was then conducted to assess the impact of these genes on the survival time of transplanted kidneys. By analyzing transcriptomics data of transplanted kidney samples from public databases, we obtained the relationship between ferroptosis-related gene (Caveolin-1, CAV1) and the prognosis of transplanted kidneys. The main function of CAV1 is to induce plasma membrane invagination, participate in cytoskeletal interactions, endocytosis, and cholesterol transport (13, 14). CAV1 is involved in stem cell differentiation, proliferation, and cell death pathways (15). Previous studies have shown that variants in the CAV1 gene are associated with post-transplant graft renal failure (16–18), but the mechanism is unclear. Subsequently, validation was carried out using clinical samples and animal models, and the underlying mechanism was explored through cell experiments, further confirming the association between high CAV1 expression and CAD.

## 2 Materials and methods

### 2.1 Data retrieval and collation

We searched the official GEO website (http://www.ncbi.nlm.nih.gov/geo/) to identify datasets related to renal transplantation and rejection. After thorough screening, we selected GSE21374 as the dataset for our study, comprising 282 expression profiles from human renal allograft biopsies. The follow-up time of each sample was extracted from the original dataset, and the samples were subsequently categorized into No-CAD and CAD groups. Data processing involved raw data download, probe annotation, imputation of missing values, and removal of differential P-values. These steps were carried out by two professional bioinformatics analysts.

### 2.2 Differential gene acquisition and prognostic modeling

Raw data from GSE21374 were log-transformed and normalized using the ‘limma’ package in R. Differential expression was identified with a threshold of log2FoldChange > 0.5. Statistical analyses were conducted using R version 4.2.2. Ferroptosis-related differential genes were identified by integrating data from the FerrDb website (http://www.zhounan.org/ferrdb/current/). Cox regression analysis was conducted utilizing the status of each kidney transplant puncture sample in GSE21374 and the corresponding follow-up time, utilizing the “survival” package. This analysis was performed using the expression matrix of ferroptosis-related genes to establish a prediction model for CAD occurrence and to derive the risk value for each sample, serving as the basis for subsequent analyses. Subsequently, target genes significantly associated with CAD occurrence were identified through the prediction model. These genes, along with the follow-up time of each sample, were utilized to conduct survival analysis on the transplant kidney survival time, facilitated by the “survival” package. This analysis aimed to further explore transplant kidney survival based on differences in time using the model established by the target genes.

### 2.3 Functional enrichment analysis

Building upon the previously obtained differentially expressed genes, we performed Gene Ontology (GO) function enrichment analysis using R language to identify the signaling pathways predominantly activated by these genes. Utilizing the risk values derived from the prediction model and the high and low expression groups of the target genes, we conducted Gene Set Enrichment Analysis (GSEA) on the gene expression matrix. This analysis identified pathways significantly enriched in the high and low-risk groups, serving as a reference for future studies. GSEA enrichment analysis was performed using the GSEA 4.1.0 software (19).

### 2.4 Clinical samples

This experiment utilized 20 transplanted kidney specimens obtained from patients diagnosed with CAD, all confirmed through histopathology at Jiangsu Provincial People’s Hospital, exhibiting interstitial fibrosis of the transplanted kidney. Exclusion criteria comprised active acute rejection, recurrence of primary renal disease (e.g., IgA nephropathy recurrence), BK virus infection, ureteral obstruction, renal malignancy, and urinary tract infection. Normal renal tissues were sourced from renal tissue specimens obtained during radical nephrectomy for renal cancer, located at least 5 centimeters (cm) from the tumor margin. The experimental protocol received approval from the Ethics Committee of Jiangsu Provincial People’s Hospital and adhered to the guidelines and regulations outlined in the Declaration of Helsinki. Informed consent was obtained from all participants.

### 2.5 Animal models

C57BL/6 and BALB/C mice were obtained from the Animal Center of Nanjing Medical University, and ethical approval for this study was obtained from the Animal Research Ethics Committee of Nanjing Medical University (Ethical code: IACUC-1805014-2). Kidneys from C57BL/6 mice were transplanted into BALB/C mice to establish a model of chronic allograft rejection (Allo group), while kidneys from BALB/C mice were transplanted into other BALB/C mice to establish a control group (Syn group). The left kidney of the donor mice was transplanted into the peritoneal cavity of the recipient mice, followed by intraoperative removal of the recipient mice’s intrinsic right kidney. At day 7 post-transplantation, the contralateral (left) kidney of the recipient mice was also removed. Intraoperatively, the mice were anesthetized with inhaled isoflurane, and the total ischemic time during surgery averaged 40 to 60 minutes. To prevent acute rejection, recipient mice were orally administered tacrolimus at a dose of 1 milligram per kilogram (mg/kg) once daily for 7 days, followed by 1 mg/kg once weekly for the next 4 weeks. At week 16 post-kidney transplantation, the mice were euthanized, and the transplanted kidney tissue was either fixed in paraffin wax or stored in liquid nitrogen (20–22). After undergoing renal transplantation, Fer-1,5 mg/kg; intraperitoneal injection was applied on postoperative days 1,3,5, to investigate the protective effects of iron death inhibitors on CAD.

### 2.6 Cell culture and processing

Cells used in this experiment were cultured in a cell culture incubator with 5% CO2 at 37°C. HK-2 cells (human proximal tubule epithelial cells) were obtained from the Cell Bank of the Chinese Academy of Sciences (Cat. SCSP-511) and cultured in DMEM/F12 medium supplemented with penicillin-streptomycin. Initially, we generated stable CAV1 knockdown or overexpression strains of HK-2 cells. Subsequently, knockdown cells were treated with 5 μM of the ferroptosis inducer (Erastin), while overexpression cells were treated with 2 μM of the ferroptosis inhibitor (Ferrostatin-1) in DMEM/F12 medium supplemented with 10% FBS for 24 hours. CAV1 knockdown, overexpression, and corresponding empty vector viruses were procured from Genechem (Shanghai, China), and cells were treated according to the manufacturer’s instructions. To establish cell co-culture systems, upper HK-2 cells (knockdown or overexpression strains) were pretreated with Erastin (5 μM, 24 hours) or Ferrostatin-1 (2 μM, 24 hours) in DMEM/F12 medium supplemented with 10% FBS for 24 hours. Subsequently, the cells were washed with PBS, the medium was replaced, and they were co-cultured with lower HK-2 cells in DMEM/F12 medium supplemented with 1% FBS for an additional 48 hours. Lysates from the lower cells were utilized for subsequent analysis.

### 2.7 Total cellular RNA extraction and polymerase chain reaction (real-time quantitative PCR, qRT-PCR)

The mRNA extraction from all samples was performed using the TRIzol method, followed by measurement of the OD value using an enzyme marker to assess RNA purity and concentration. Subsequently, samples were stored at -80°C. RNA samples were reverse transcribed using HiScript^®^ III All-in-one RT SuperMix (Vazyme, R333-01) according to the manufacturer’s instructions. qPCR assays were conducted using ChamQ SYBR Color qPCR Master Mix (Vazyme, Q431-02) according to the manufacturer’s protocol. Upon completion of the reaction program, the relative expression levels of different genes in the samples were calculated based on the Ct values obtained. Primer sequences used for qPCR are provided in **Supplementary File 1**.

### 2.8 Western blot

Sample proteins were extracted and their concentrations were determined. Subsequently, the samples were loaded into the wells of an electrophoresis comb tank. Electrophoresis was performed at a constant voltage of 60V initially, followed by increasing the voltage to 120V after separation of the marker on the concentrated gel surface. Electrophoresis was halted once the protein marker reached the appropriate position on the separated gel. 1× transfer buffer was prepared, and the PVDF membrane was cut to match the size of the gel. The PVDF membrane was activated using methanol and subsequently subjected to membrane transfer under constant current at 300mA. Following 2 hours of blocking with skimmed milk, antibody incubation was conducted overnight at 4°C. The membrane was washed three times for 15 minutes each with 1× TBST solution. Subsequently, the membrane was incubated with the horseradish peroxidase-coupled secondary antibody with gentle shaking at room temperature for 2 hours. Exposure solution A and solution B were mixed in a 1:1 ratio under light protection. The resulting exposure solution was evenly applied onto the PVDF membrane, covered with a transparent membrane, and exposed using a Bio-RAD exposure meter. The results were visualized using Image Lab software and saved. Information on antibodies and their dilution ratios can be found in **Supplementary File 2**.

### 2.9 Immunohistochemistry, immunofluorescence, hematoxylin and eosin, and Masson staining

Renal tissues were fixed with 4% paraformaldehyde and embedded in paraffin. Three-micrometer-thick sections were obtained and baked at 65℃ for 120 min, then deparaffinized with xylene and rehydrated in graded ethanol (100%, 100%, 95%, 85%, and 75% separately). Heat-induced antigen unmasking was performed with Sodium Citrate Antigen Retrieval Solution (Solarbio, C1032) or EDTA Antigen Retrieval Solution (Solarbio, C1034). Immunostaining was then blocked with block buffer (Beyotime, P0260) for 30 min at room temperature, followed by overnight incubation with primary antibodies in a humidified chamber at 4°C overnight. Primary antibodies applied in IHC were (CAV1(Proteintech,16447-1-AP,1:500); GPX4 (Abcam, ab125066, 1:500); TFR (Proteintech, 66180-1-Ig,1:500); Secondary antibodies from the IHC secondary antibody Kit (Absin, abs996) were incubated at room temperature for 60 min. Sections were stained with diaminobenzidine (DAB) and counterstained with hematoxylin. Primary antibodies applied in IF were (CAV1(Proteintech,16447-1-AP,1:500); GPX4 (Abcam, ab125066, 1:500); TFR (Proteintech, 66180-1-Ig,1:500); Secondary antibodies were Alexa Fluor 488- Conjugate Anti-Rabbit IgG (H+L) (CST, 4412S, 1:500); Alexa Fluor 594- Conjugate Anti- Mouse IgG (H+L) (CST, 8890S, 1:500)), the nuclei were stained using 4’,6-diamidino-2-phenylindole(DAPI), after that the slides were sealed. Kidney tissue sections were stained with HE and Masson for histopathological examination was performed as previously described (23).

### 2.10 RNS detection

Based on the results of pathway enrichment and previous research indicating that CAV1 can participate in the regulation of RNS, we will proceed to detect RNS in various cell experiments (24, 25). The levels of reactive nitrogen species (RNS) were assessed using an active nitrogen detection kit (Bestbio, China, BB-462112) according to the manufacturer’s instructions.

### 2.11 Enzyme-linked immunosorbent assay

The levels of Interleukin-1β (IL-1β), Transforming Growth Factor-β1 (TGF-β1), Platelet-Derived Growth Factor-BB (PDGF-BB), Interleukin-6(IL-6), and tumor necrosis factor-α (TNF-α) in the cell culture medium were assessed using IL-1β (mlbio, China, YJ058059), TGF-β1 ((mlbio, China, YJ022522), PDGF-BB ((mlbio, China, YJ023009), IL-6 ((mlbio, China, YJ028583), and TNF-α (Elabscience, China, E-EL-H0109) ELISA kits according to the manufacturer’s instructions.

### 2.12 Statistical analysis

The data were analyzed using R 4.2.2. Data were presented as means ± Standard Error of Measurement (SEM). Statistical comparisons between the two groups were performed utilizing Student’s t-test, and multiple comparisons were conducted applying one-way ANOVA with *post-hoc* Tukey’s or Dunnett’s multiple comparison tests or two-way ANOVA with *post-hoc* Tukey’s multiple comparisons test. The data was statistically analyzed using SPSS 26.0 software (SPSS Inc, USA). Use GraphPad software (8.0.2) to draw and analyze statistical charts, and the P-value is less than 0.05. *, P < 0.05; **, P < 0.01; ***, P < 0.001; ns, non-significant.

## 3 Results

### 3.1 High expression of CAV1 is positively correlated with poor prognosis in CAD patients

**Figure 1:**
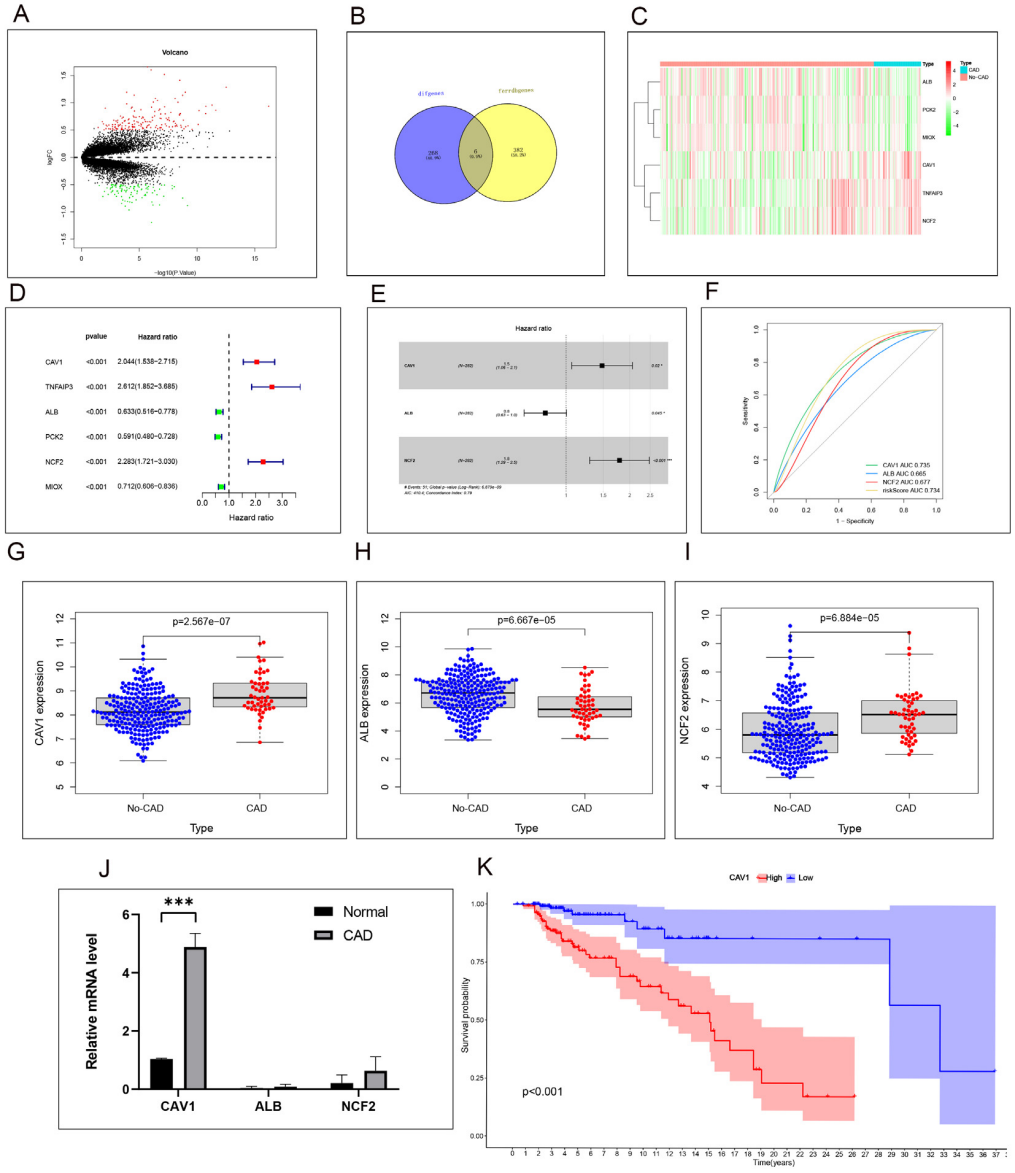
High expression of CAV1 is positively correlated with poor prognosis in CAD patients. **(A)** Volcano plot of differentially expressed genes (DEGs) in CAD vs. non-CAD samples. **(B)** Intersection of ferroptosis-related and DEGs identified six key genes. **(C)**Heat map analysis of 6 differential ferroptosis-related genes expressions between the CAD and No-CAD groups. **(D)**:Uni-factor Cox regression analysis of six target genes in the CAD and No-CAD groups, the Hazard ratio values of the red point are greater than 1, and the Hazard ratio values of the green point are less than 1. **(E)** multi-factor Cox regression analysis of six target genes in the CAD and No-CAD groups, the Hazard ratio values of CAV1 and NCF2 is greater than 1, and the Hazard ratio values of ALB is less than 1,P<0.05. **(F)** ROC curves showing AUC values for CAV1, ALB, and NCF2 in predicting CAD. The AUC values reflect the predictive power of CAV1, ALB, and NCF2 for CAD. **(G–I)** Results of the comparative analysis of the expression of the three target genes in the CAD and No-CAD groups. The expression of CAV1 and NCF2 in the CAD group was significantly higher than that in the No-CAD group, while the expression of ALB in the CAD group was significantly lower than that in the No-CAD group, with all P <0.05. **(J)** The relative mRNA level of CAV1,ALB and NCF2 in renal tissue from the normal and CAD groups was detected by qPCR, the most significant differences in the expression of CAV1. **(K)** Results of survival time or follow-up time survival analysis of transplanted kidneys based on the expression of CAV1. *P < 0.05, *** P < 0.001.

We analyzed the differentially expressed genes in CAD and No-CAD samples from the dataset GSE21374, resulting in the identification of 274 differentially expressed genes. Specifically, 171 genes exhibited high expression levels in CAD tissues, whereas 103 genes showed low expression levels in CAD tissues (**Figure 1A**). We then retrieved ferroptosis-related genes from the FerrDb website, resulting in the identification of 388 genes associated with ferroptosis, including driver, suppressor, and marker genes. These genes were intersected with the differentially expressed genes from dataset GSE21374, yielding six differential genes related to ferroptosis: Caveolin-1 (CAV1), Albumin (ALB), Neutrophil cytosolic factor 2 (NCF2), TNF-α-induced protein 3 (TNFAIP3), Phosphoenolpyruvate Carboxykinase 2 (PCK2), and Myo-Inositol Oxygenase (MIOX). These genes exhibited differential expression between the CAD and No-CAD groups (**Figure 1B**). Subsequently, we isolated the expression profiles of ferroptosis-related genes exhibiting differential expression in the CAD and No-CAD groups, followed by a comparative analysis. The outcomes of this analysis are visualized in the heat map (**Figure 1C**). Initially, we conducted a univariate Cox analysis integrating the expression profiles of differential genes from GSE21374, sample status, and follow-up durations for each sample. Univariate Cox regression analysis revealed the relationships between 6 target genes (CAV1, TNFAIP3, ALB, PCK2, NCF2, and MIOX) and CAD (**Figure 1D**), while multi-factor Cox regression analysis revealed the relationships between 3 target genes (CAV1, ALB, and NCF2) and CAD(**Figure 1E**). The specific Hazard ratio and P-value results are provided in **Supplementary File 3**. Following this, we evaluated the ROC results of the models constructed by our three target genes. Both the individual genes and the combined risk score exhibited strong predictive capabilities: CAV1 (AUC value: 0.735, 95% CI 0.665-0.805), ALB (AUC value: 0.665, 95% CI 0.582-0.744), NCF2 (AUC value: 0.677, 95% CI 0.609-0.739), and the risk score (AUC value: 0.734, 95% CI 0.668-0.796) (**Figure 1F**). Among CAV1, ALB, and NCF2, CAV1 has the largest area under the ROC curve. The AUC values reflect the predictive power of CAV1, ALB, and NCF2 for CAD. The expression levels of the three modeling genes were extracted, revealing increased expression levels of CAV1 and NCF2 in the CAD group, whereas the expression levels of ALB decreased in the CAD group (**Figures 1G–I**). mRNA was extracted and the relative expression levels of CAV1, ALB, and NCF2 were validated using qRT-PCR. Specifically, the results of CAV1 were consistent with those of GSE21374, demonstrating the most significant difference (**Figure 1J**). Based on the validation results of the previous ROC curve, we chose to focus on CAV1 as the next research objective. Account for confounders of the use/follow-up time of transplanted kidneys, whether the renal function has failed, and whether rejection reactions have occurred, analysis of the Kaplan-Meier curve revealed a significant difference in the duration of transplanted kidney usage between the high and low expression groups of CAV1, with a p-value less than 0.05 (**Figure 1K**).

### 3.2 Increased expression of CAV1 in CAD tissues compared to the control group in clinical samples

**Figure 2:**
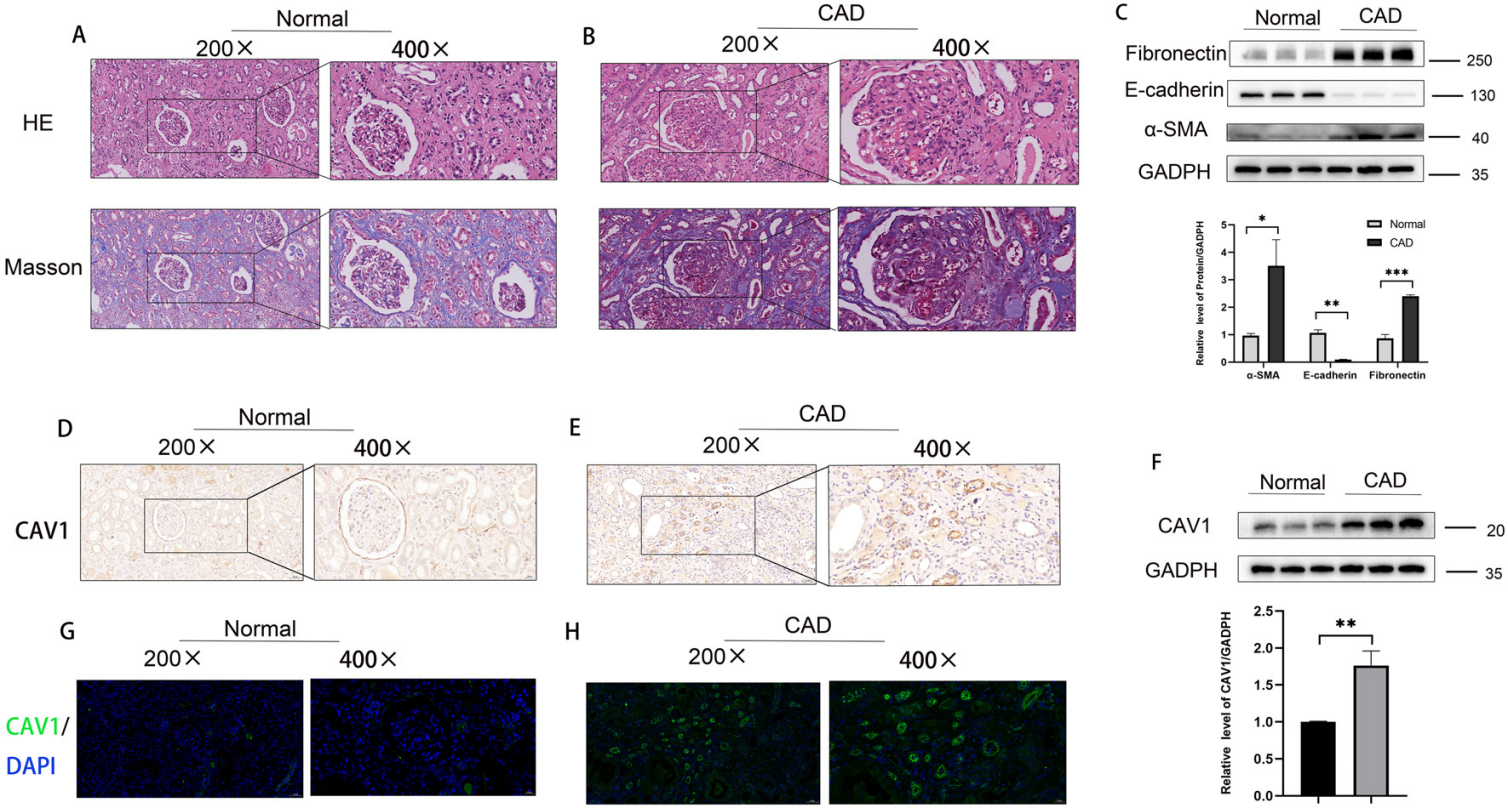
Increased expression of CAV1 in CAD tissues compared to the control group in clinical samples. **(A)** HE and MASSO staining results of the control group, **(B)** HE and MASSO staining results of the CAD group. **(C)** Protein levels of E-cadherin, α-SMA and Fibronectin in renal tissue from the normal and CAD groups were analyzed by western blot assay. *P < 0.05, **P < 0.01, ***P < 0.001 **(D, E)** Representative IHC images of CAV1 expression in renal tissue from normal and CAD groups. **(F)** Protein levels of CAV1 in renal tissue from the normal and CAD groups were analyzed by western blot assay. **P < 0.01. **(G, H)** Representative immunofluorescence images of CAV1 expression in renal tissue from normal and CAD groups.

Samples were classified into a control group and a CAD group following clinical source analysis. The results of HE and MASSO staining showed a higher severity of tubular atrophy, damage, and renal fibrosis in the kidneys of the CAD group in comparison to the control group (**Figures 2A, B**). Furthermore, Western blot experiments comparing the control and CAD groups exhibited a notable decrease in E-cadherin and a substantial increase in α-SMA and Fibronectin levels (**Figure 2C**). To provide further validation of our observations, we assessed the expression disparities of CAV1 between the control and CAD groups through immunohistochemistry. The outcomes demonstrated substantial expression of CAV1 in the atrophic renal tubules (**Figures 2D, E**), which was supported by immunofluorescence results (**Figures 2G, H**). The Western blot results revealed a notable disparity in the protein levels of CAV1 in CAD renal tissue compared to the control group (**Figure 2F**).

### 3.3 Increased expression of CAV1 in the ALLO group compared to the SYN group in animal models

**Figure 3:**
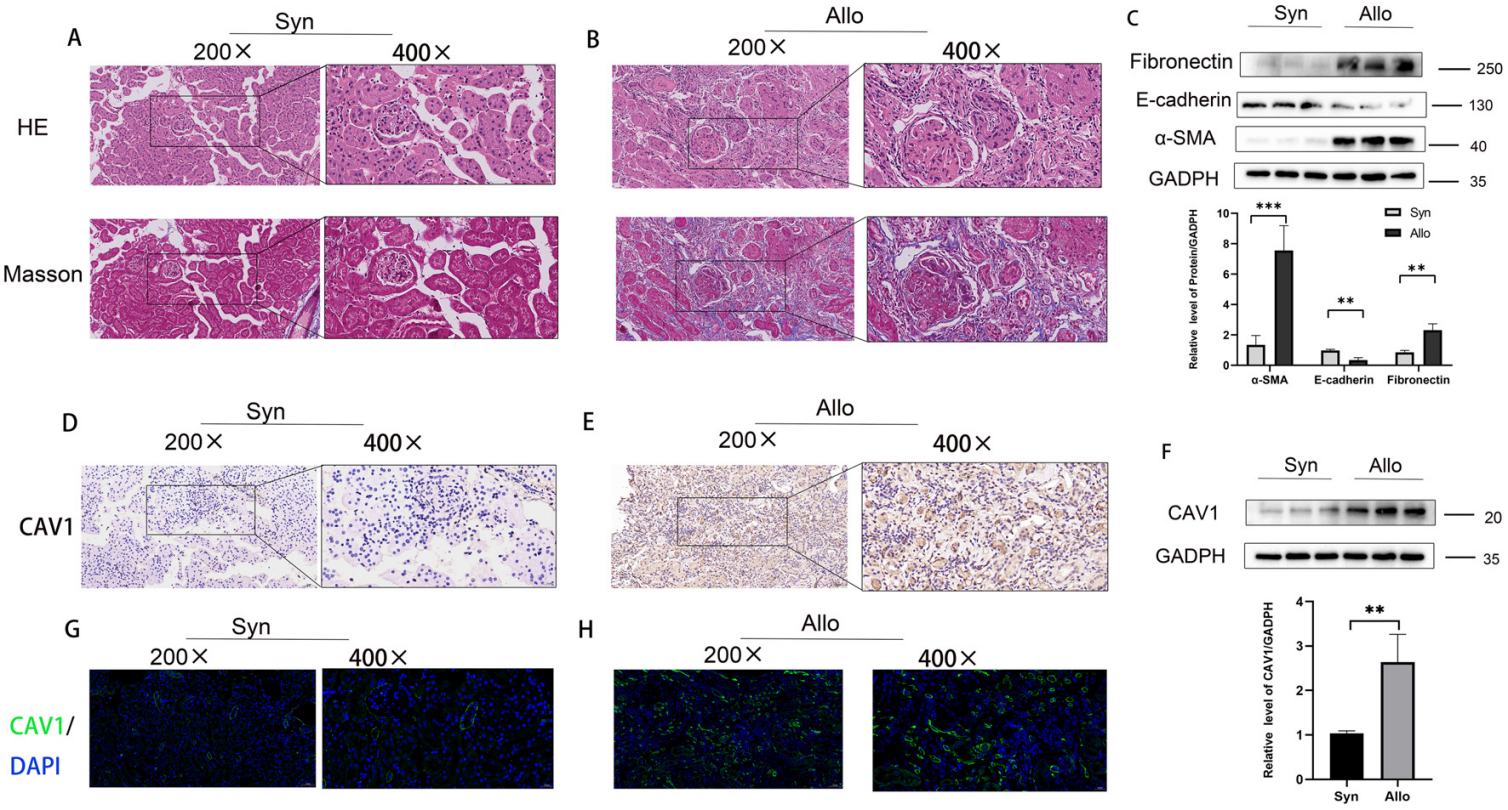
Increased expression of CAV1 in the ALLO group compared to the SYN group in animal models. **(A)** HE and MASSO staining results of the Syn group, **(B)** HE and MASSO staining results of the Allo group. **(C)** Protein levels of E-cadherin, α-SMA and Fibronectin in renal tissue from the Syn and Allo groups were analyzed by western blot assay. **P < 0.01, ***P < 0.001. **(D, E)** Representative IHC images of CAV1 expression in renal tissue from Syn and Allo groups. **(F)** Protein levels of CAV1 in renal tissue from the Syn and Allo groups were analyzed by western blot assay. **P < 0.01. **(G, H)** Representative immunofluorescence images of CAV1 expression in renal tissue from Syn and Allo groups.

An animal model of chronic rejection of kidney transplantation was established in mice, and the results are depicted in **Supplementary Figures 2A, B**. Consistent with observations from previous clinical samples, the 16-week mouse kidney transplantation model displayed heightened renal fibrosis, tubular damage, and atrophy in the ALLO group in comparison to the SYN group (**Figures 3A, B**). Indicators of fibrosis were detected in the mouse kidney transplantation model via WB analysis, revealing a significant decrease in E-cadherin and a significant increase in α-SMA and Fibronectin levels (**Figure 3C**). To validate our observations, we assessed the disparity in CAV1 expression between the SYN and ALLO groups using immunohistochemistry and immunofluorescence. The outcomes demonstrated predominant expression of CAV1 in atrophic renal tubules, consistent with the findings observed in the clinical samples (**Figures 3D, E, G, H**). The Western blot results exhibited a notably higher protein level of CAV1 in the ALLO group in comparison to the SYN group (**Figure 3F**).

### 3.4 Activation of ferroptosis pathway in transplanted kidney tissue with chronic rejection

**Figure 4:**
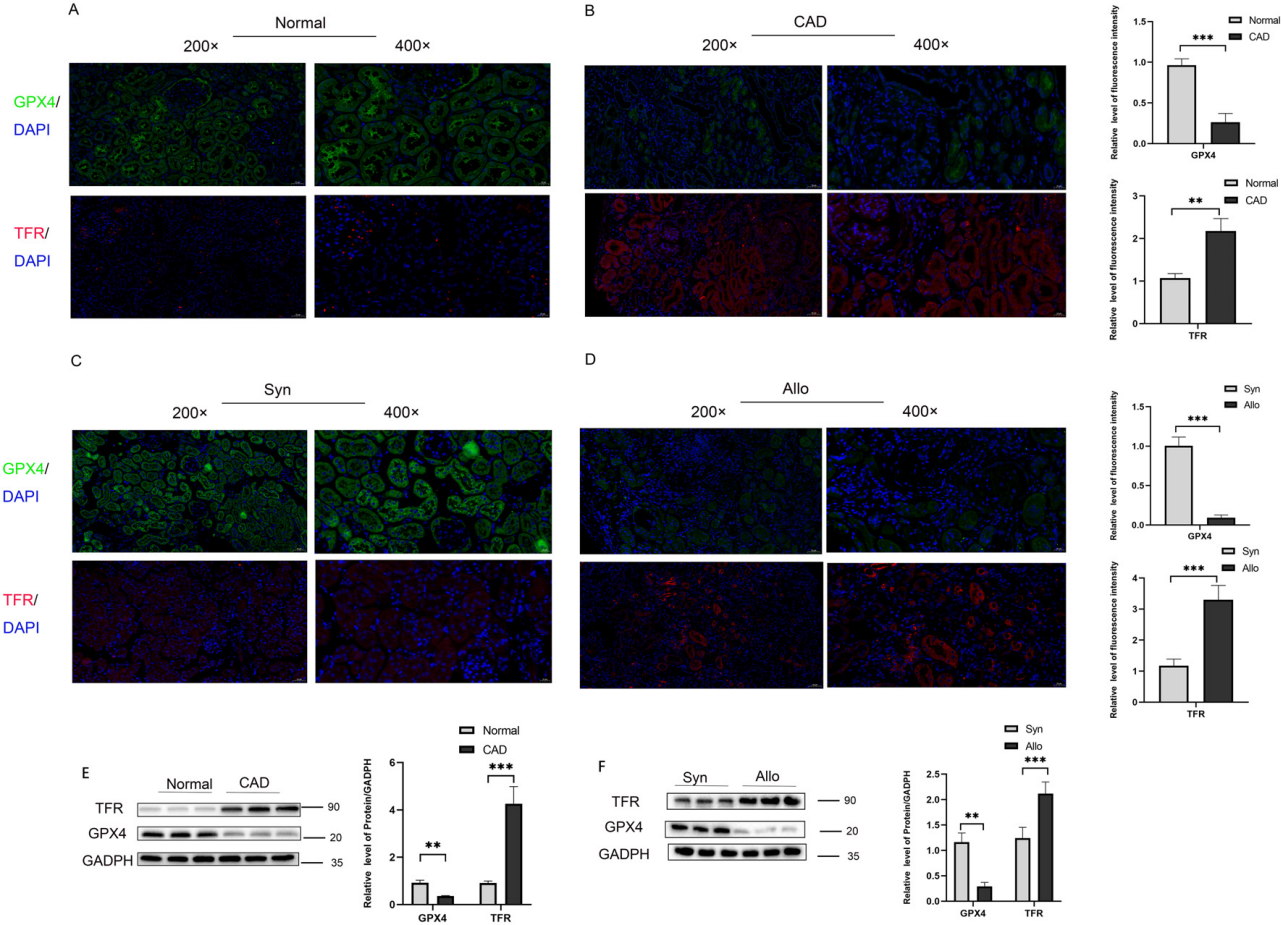
Activation of ferroptosis pathway in transplanted kidney tissue with chronic rejection **(A)** Representative immunofluorescence images of GPX4 (green) and TRF (red) expression in renal tissue from normal group, **(B)** Representative immunofluorescence images of GPX4 (green) and TRF (red) expression in renal tissue from CAD group. **(C)** Representative immunofluorescence images of GPX4 (green) and TRF (red) expression in renal tissue from Syn group, **(D)** Representative immunofluorescence images of GPX4 (green) and TRF (red) expression in renal tissue from Allo group. **(E)** Protein levels of GPX4 and TFR in renal tissue from the Normal and CAD groups were analyzed by western blot assay. *P < 0.05, **P < 0.01, ***P < 0.001. **(F)** Protein levels of GPX4and TFR in renal tissue from the Syn and Allo groups were analyzed by western blot assay. *P < 0.05, **P < 0.01, ***P < 0.001.

Furthermore, analysis of the two markers of ferroptosis, Glutathione Peroxidase 4 (GPX4) and transferrin receptor (TFR) reveals significant activation of the ferroptosis pathway in CAD development, evidenced by a significant decrease in GPX4 and a significant increase in TFR levels **(Figures 4A, B**). To further validate our conclusion, we assessed these two markers through immunofluorescence in a mouse animal model and obtained consistent results with those observed in clinical samples (**Figures 4C, D**). These results are consistent with our previous research: the ferroptosis signaling pathway is activated in CAD tissues (23). The results of western blot showed a significant decrease in the level of GPX4 and a significant increase in the level of TFR in CAD tissues compared to the control group (**Figure 4E**), and the same results were obtained in a mouse kidney transplantation animal model (**Figure 4F**). With the occurrence of ferroptosis of tubular epithelial cells in the transplanted kidney tissues, the damaged cells secrete a large amount of inflammatory factors to aggravate the inflammatory response and interstitial fibrosis of the transplanted kidney, accelerating the progression of CAD.

### 3.5 The expression of CAV1 impacts the ferroptosis pathway

**Figure 5:**
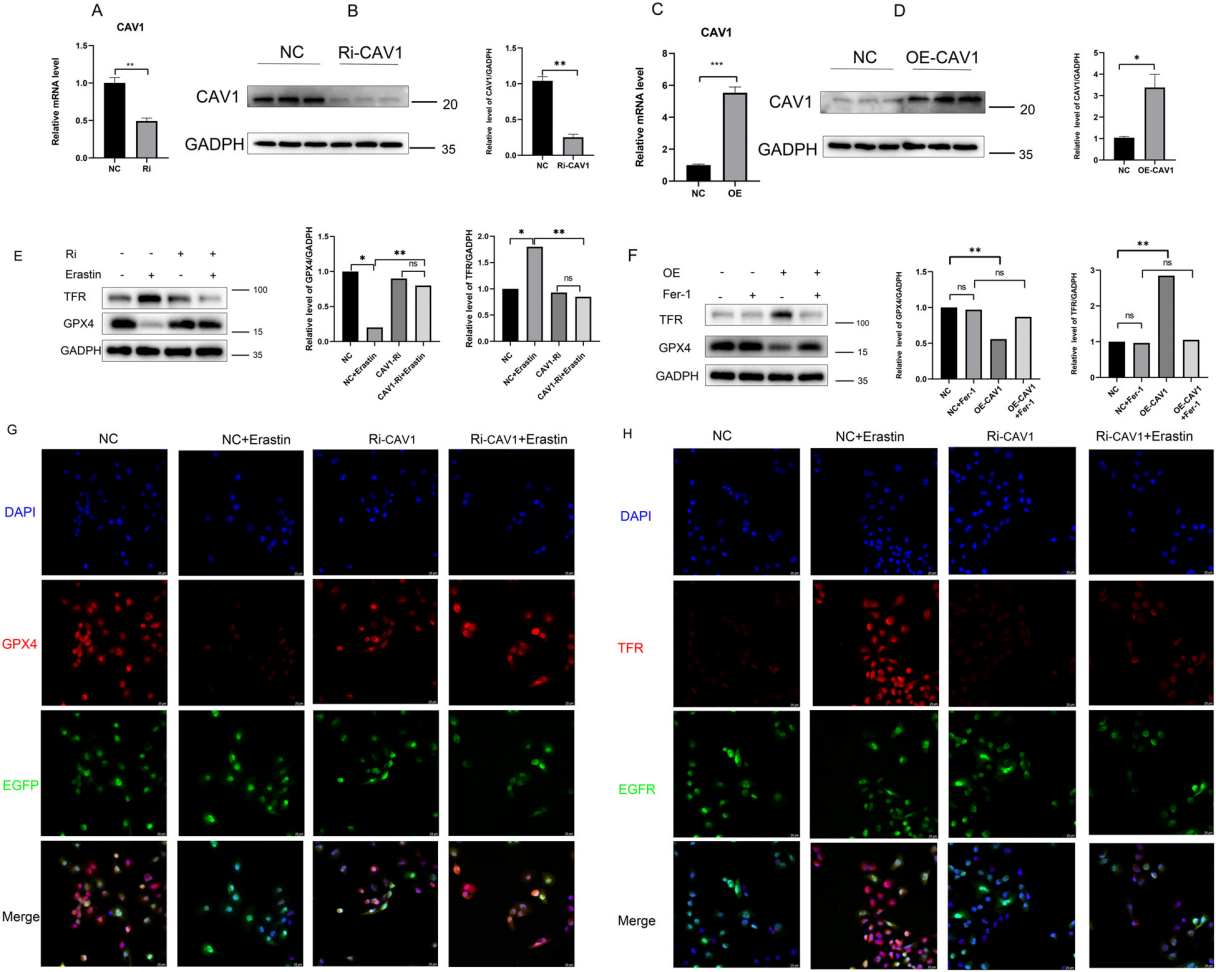
The expression of CAV1 impacts the ferroptosis pathway **(A)** The relative mRNA level of CAV1 in cell culture from the control groups(NC) and CAV1 knockdown groups (Ri-CAV1) were detected by qPCR, **P < 0.01. **(B)** Protein levels of CAV1 in cell culture from the control groups (NC) and CAV1 knockdown groups (Ri-CAV1) were analyzed by western blot assay, **P < 0.01. **(C)** The relative mRNA level of CAV1 in cell culture from the control groups (NC) and CAV1 overexpression groups (OE-CAV1) were detected by qPCR, **P < 0.01. **(D)** Protein levels of CAV1 in cell culture from the control groups (NC) and overexpression groups (OE-CAV1) were analyzed by western blot assay,**P < 0.01. **(E)** Protein levels of GPX4 and TFR were analyzed by western blot assay in HK-2 cells (control groups (NC) or CAV1 knockdown groups (Ri-CAV1)), treated with Erastin (5 μM). **(F)** Protein levels of GPX4 and TFR were analyzed by western blot assay in HK-2 cells [control groups (NC) and CAV1 overexpression groups (OE-CAV1)], treated with Ferrostatin-1 (2 μM). **(G)** Representative immunofluorescence images of GPX4 expression in HK-2 cells from the control groups (NC) and CAV1 knockdown groups (Ri-CAV1). **(H)** Representative immunofluorescence images of TFR expression in HK-2 cells from the control groups (NC) and CAV1 knockdown groups (Ri-CAV1). Blue: DAPI; Red: GPX4 or TFR; Green: Enhanced Green Fluorescent Protein (EGFP), Green fluorescence after cell transfection with lentivirus.

To further explore the mechanism through which differential CAV1 expression affects CAD occurrence in transplanted kidneys via the ferroptosis pathway, we performed *in vitro* cell experiments. We successfully generated CAV1 knockdown and overexpression HK2 cell lines using lentivirus and confirmed the efficiency of knockdown and overexpression, as depicted in the cell fluorescence diagram in **Supplementary Figure 2** (knockdown group: C-D; overexpression group: E-F). Compared to the empty virus, the knockdown efficiency of the knockdown group exceeded 50%, while the efficiency of the overexpression group doubled. qRT-PCR and WB analyses validated the mRNA and protein levels of CAV1 (**Figures 5A–D**). Following Erastin induction, GPX4 markedly decreased and TFR substantially increased in the control group. In the CAV1 knockdown group, GPX4 and TFR levels did not significantly differ from those in the control group. Under CAV1 knockdown, there was no significant change in these two ferroptosis markers, even under Erastin induction (**Figure 5E**). In the CAV1 overexpression group, it was observed that as the expression level of CAV1 increased, GPX4 significantly decreased and TFR significantly increased compared to the control group. Additionally, under the action of Ferrostatin-1, there was no significant change in these two ferroptosis markers (**Figure 5F**). Similarly, consistent outcomes were obtained through cellular immunofluorescence analysis (**Figures 5G, H**). This part of the results indicates that the expression of CAV1 impacts the ferroptosis pathway.

### 3.6 RNS exhibits significant differences in CAV1-knockdown and CAV1-overexpression groups

**Figure 6:**
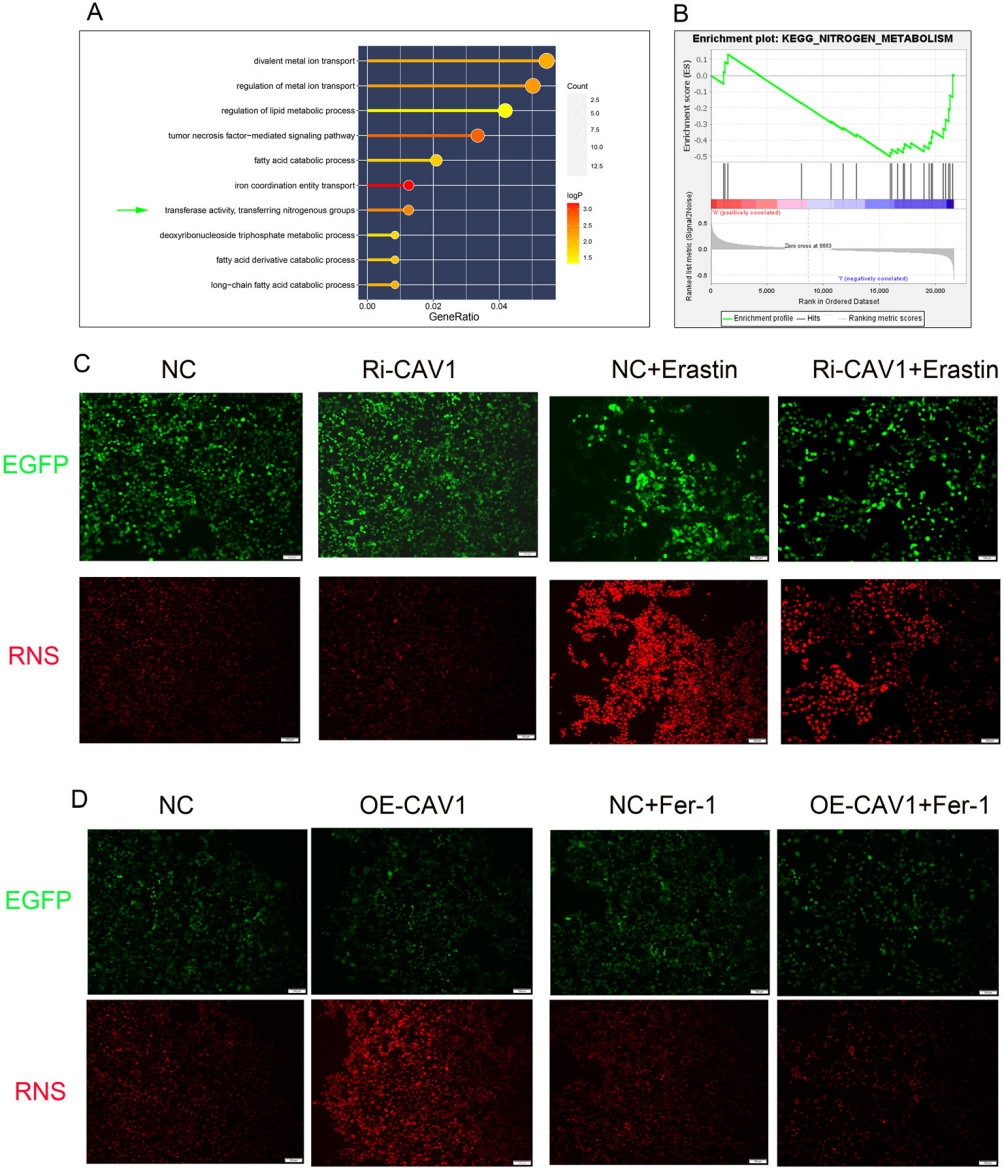
RNS exhibits significant differences in CAV1-knockdown and CAV1-overexpression groups **(A)** The results of GO functional enrichment analysis, the size of the dots represents the number of genes enriched on each pathway and the color of the dots represents the P value. **(B)** The results of GSEA functional enrichment analysis, the peak of the curve below the horizontal line represents that the pathway is actively expressed in the low-risk group. **(C)** Representative immunofluorescence images of RNS expression in HK-2 cells from the control groups (NC) and CAV1 knockdown groups (Ri-CAV1) treated with Erastin (5 μM). **(D)** Representative immunofluorescence images of RNS expression in HK-2 cells from the control groups (NC) and CAV1 overexpression groups (OE-CAV1)), treated with Ferrostatin-1 (2 μM). Red: RNS; Green: Enhanced Green Fluorescent Protein (EGFP).

The GO functional enrichment analysis revealed significant activation of the “transferase activity, transferring nitrogenous groups” pathway (**Figure 6A**). Likewise, the GSEA pathway enrichment analysis demonstrated significant enrichment of the “KEGG-NITROGEN-METABOLISM” pathway (**Figure 6B**). Within the CAV1 knockdown group of cells, there was no significant change in the level of RNS compared to the control group. However, upon Erastin induction, the expression level of RNS significantly increased in the control group. Notably, in the CAV1 knockdown group, the increase in RNS was mitigated (**Figure 6C**). In the CAV1 overexpression group of cells, the expression level of RNS was notably higher compared to the control group. However, with the influence of Ferrostatin-1, there was no notable difference in the level of RNS between the control and overexpression groups (**Figure 6D**).

#### 3.6.1 CAV1 promotes EMT in HK2 cells through ferroptosis pathway

**Figure 7:**
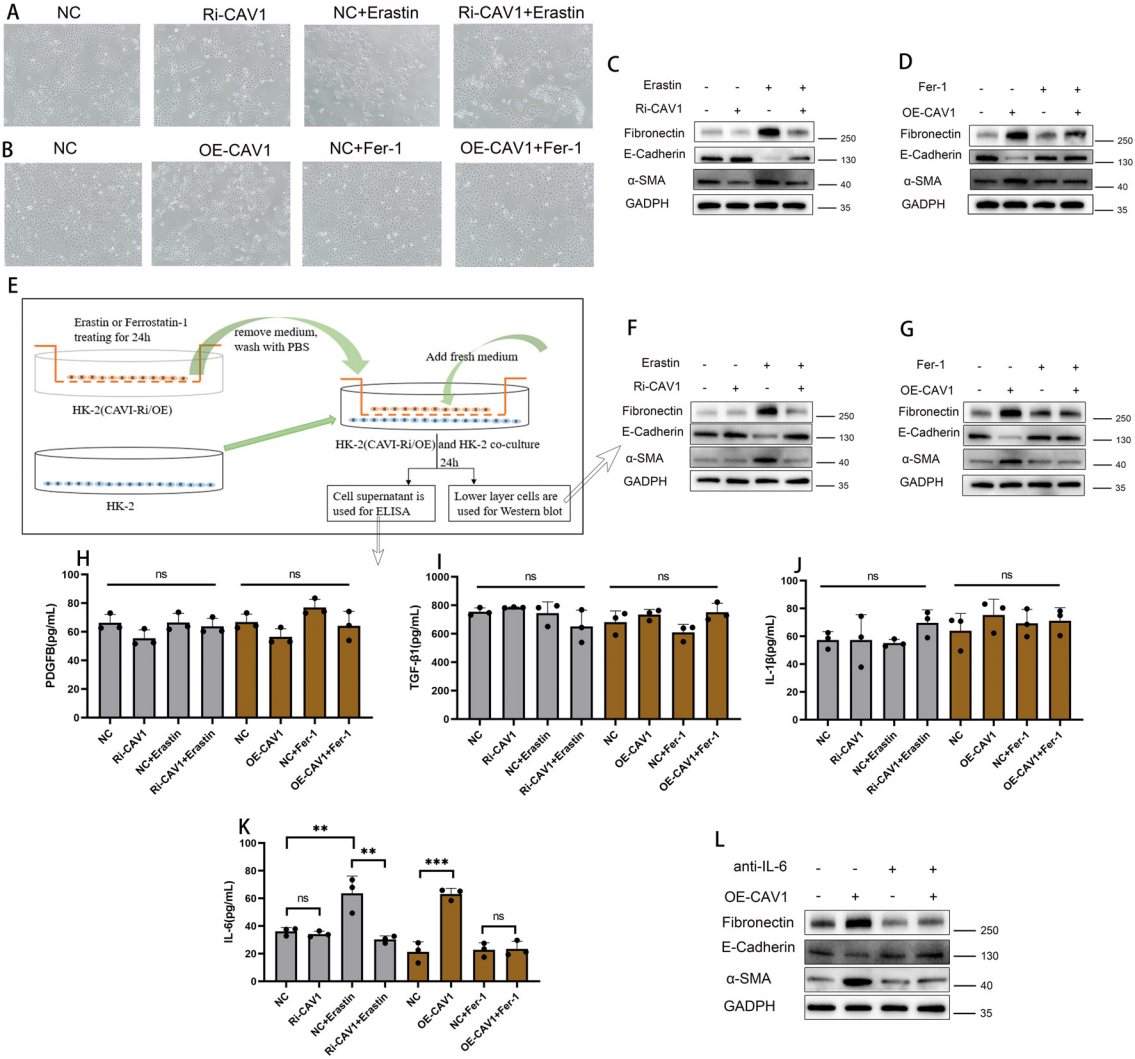
CAV1 secretes pro-fibrotic cytokines (IL-6) through the ferroptosis pathway, promoting EMT in HK2 cells **(A)** Optical microscopy examination of the HK2 cell lines from the control groups (NC) and CAV1 knockdown groups(Ri-CAV1) treated with Erastin (5 μM). **(B)** Optical microscopy examination of the HK2 cell lines from the control groups (NC) and CAV1 overexpression groups (OE-CAV1)), treated with Ferrostatin-1 (2 μM). **(C)** Protein levels of E-cadherin, α-SMA, and Fibronectin in cell culture from the control groups (NC) and CAV1 knockdown groups (Ri-CAV1) treated with Erastin (5 μM) were analyzed by western blot assay. **(D)** Protein levels of E-cadherin,α-SMA,and Fibronectin in cell culture from the control groups (NC) and CAV1 overexpression groups (OE-CAV1)), treated with Ferrostatin-1 (2 μM) were analyzed by western blot assay. **(E)** Flowchart of cell co-culture. **(F)** Protein levels of E-cadherin, α-SMA and Fibronectin in HK-2 cells from the co-culture (control groups (NC) and CAV1 knockdown groups (Ri-CAV1) treated with Erastin (5 μM)). **(G)** Protein levels of E-cadherin, α-SMA, and Fibronectin in HK-2 cells from the co-culture(control groups (NC) and CAV1 overexpression groups (OE-CAV1)), treated with Ferrostatin-1 (2 μM)). **(H–J)** Levels of PDGF-BB, TGF-β1, and IL-1β in the cell culture medium of co-culture models were analyzed by ELISA assay. **(K)** Levels of IL-6 in the cell culture medium of co-culture models were analyzed by ELISA assay. NS (no significance), ** P < 0.01, *** P < 0.001. **(L)** Protein levels of E-cadherin, α-SMA, and Fibronectin in HK-2 cells from the co-culture(control groups (NC) and CAV1 overexpression groups (OE-CAV1)), treated with neutralizing antibodies of IL-6).

Optical microscopy examination of the HK2 cell line in the CAV1 knockdown group revealed no significant alteration in cell morphology compared to the control group. However, upon Erastin stimulation, a more pronounced change in cell state was observed in the control group, with some cells exhibiting noticeable signs of death. In contrast, the CAV1 knockdown group showed significant mitigation of cell death (**Figure 7A**). Optical microscopy analysis of the HK2 cell line in the CAV1 overexpression group revealed significant clustering of cells in morphology compared with the control group. Cells transitioned from a cobblestone-like appearance to spindle-shaped, indicative of epithelial-mesenchymal transition (EMT) and some cell death. However, under the influence of Ferrostatin-1, no significant change was observed between the CAV1 overexpression group and the control group (**Figure 7B**). Western blot analysis revealed that Erastin treatment led to a decrease in epithelial cell markers (E-cadherin) observed in the control group, while fibrosis indicators (α-SMA and Fibronectin) increased. However, in the CAV1 knockdown group, there were no significant changes in these indicators even under Erastin induction (**Figure 7C**). In contrast, the CAV1 overexpression group exhibited a reduction in epithelial cell markers and an elevation in fibrosis markers compared to the control group. Moreover, under the influence of Ferrostatin-1, there was no significant alteration in these indicators between the CAV1 overexpression group and the control group (**Figure 7D**). This part of the results indicates that CAV1 promotes EMT in HK2 cells through ferroptosis pathway. However, following cell death, the process of EMT cannot proceed. We hypothesize that this may be attributed to the secretion of specific pro-fibrotic factors by cells undergoing ferroptosis, consequently influencing the EMT process in surrounding cells.

#### 3.6.2 CAV1 secretes pro-fibrotic cytokines (IL-6) through the ferroptosis pathway, promoting EMT in HK2 cells

In cell culture, certain cells undergo cell death while others undergo epithelial-mesenchymal transition (EMT). To investigate this phenomenon, we employed a co-culture model (**Figure 7E**). In the co-culture experiment, WB analysis of the lower layer cells revealed that in the CAV1 knockdown group, Erastin induced significant changes in EMT indicators compared to the control group (increase in α-SMA and Fibronectin; decrease in E-cadherin). However, in the CAV1 knockdown group, these indicators of the lower layer cells did not exhibit significant changes (**Figure 7F**). In the CAV1 overexpression group, significant changes were observed in EMT indicators (increase in α-SMA and Fibronectin; decrease in E-cadherin). However, under the influence of Ferrostatin-1, these indicators did not exhibit significant changes (**Figure 7G**). The ELISA assay was employed to detect common pro-fibrotic factors in the cell culture medium of the co-culture model. The results revealed no significant difference in the changes of PDGF-BB, IL-1β, and TGF-β (**Figures 7H–J**), and the concentration of TNF-α was below the detection limit of the reagent kit, hence not shown. However, notable differences were observed in the concentration of IL-6 (**Figure 7K**). In our present study, we found that CAV1 overexpression could promote the occurrence of epithelial cell ferroptosis, which is an inflammatory mode of death, and IL-6 is also mainly involved in the inflammatory response, as a result, IL-6 levels were increased after CAV1 overexpression. To further validate the role of IL-6, neutralizing antibodies against IL-6 were added to co-cultured cell media overexpressing CAV1. Subsequent WB analysis of lower layer cells revealed that the changes in EMT were significantly reduced under the action of IL-6 neutralizing antibodies (**Figure 7L**).

## 4 Discussion

Allogeneic kidney transplantation stands as the optimal kidney replacement therapy for end-stage renal disease (26, 27). This is attributed to its significant enhancement of the quality of life for patients with end-stage renal disease. However, acute and chronic graft rejection subsequent to kidney transplantation substantially curtails its efficacy, thereby inducing considerable distress for both patients and clinicians (28). The diagnosis of graft rejection currently depends on the pathological biopsy report, which entails an invasive procedure that could lead to post-puncture renal bleeding and exacerbate the loss of renal function (29). In recent years, owing to the continuous advancement in gene sequencing technology, there has been a growing interest in the genetic and molecular aspects of rejection in kidney transplantation and post-transplantation. However, the molecular mechanisms underlying acute and chronic rejection after kidney transplantation remain unclear. The process of epithelial-mesenchymal transition (EMT) leads to significant intercellular material deposition, representing a key process in chronic renal fibrosis, extensively studied in conditions such as hypertensive nephropathy and chronic kidney disease (30, 31). Our study is centered on investigating the impact of EMT on chronic transplant renal fibrosis and exploring strategies to mitigate EMT progression, thus prolonging the functionality of transplanted kidneys.

Ferroptosis, characterized by iron-dependent regulated cell death, arises from the accumulation of reactive oxygen species (ROS), initiating a cascade of intracellular oxidative events culminating in cell lysis and demise (7). The concept of ferroptosis has garnered significant attention since its inception. Previous studies suggest that ferroptosis contributes substantially to drug resistance in tumor cells, ischemic organ damage, and various degenerative diseases associated with extensive lipid peroxidation (32). Criteria for iron-dependent cell death include an imbalance in cellular metabolism and redox homeostasis, leading to lethal lipid peroxidation and subsequent cell demise (33, 34). Anemia and disrupted iron homeostasis are prevalent in chronic kidney disease and are associated with serious adverse consequences (35). Iron supplementation is commonly employed in treating renal anemia. Factors such as iron ion overload in renal tubular epithelial cells and the microenvironment of chronic inflammation in transplanted kidneys contribute to ferroptosis (36). These factors motivate our investigation into iron-dependent cell death in chronic transplanted renal fibrosis. Not all cells undergo ferroptosis during chronic rejection of transplanted kidneys (37, 38), similar to the variation observed in renal tubular atrophy and fibrosis on pathological sections (39, 40). Ferroptosis plays an important role in cardiomyocyte injury and can be induced by plasma peroxidation products (41). Studies have shown that increased ferroptosis after myocardial ischemia, cardiomyopathy, and myocardial infarction may lead to cardiomyocyte death, which can exacerbate the fibrotic process (42). In diseases such as nonalcoholic fatty liver disease (NAFLD) and alcoholic liver disease, ferroptosis may lead to disorders of lipid metabolism and cellular damage (43, 44). Activation and proliferation of fibroblasts are critical steps in the process of liver fibrosis. Ferroptosis can promote fibrosis by activating fibroblasts through increased oxidative stress and decreased antioxidant defenses (45). Ferroptosis may result in damage to alveolar epithelial cells and fibroblasts, increasing the production and release of inflammatory mediators to exacerbate the inflammatory response in pulmonary fibrosis (46, 47). In the context of chronic inflammation, certain renal tubules experience iron-dependent cell death, resulting in cell lysis, extravasation of cellular contents, and release of significant amounts of pro-fibrotic factors, which influence neighboring cells and potentially contribute to epithelial-mesenchymal transition (EMT), thereby accelerating the progression of transplantation renal fibrosis (48, 49). Our objective is to investigate the mechanism of ferroptosis during the chronic allograft dysfunction (CAD) process.

Caveolin-1 (CAV1) plays a significant role in signaling receptor binding, response to hypoxia, protein kinase binding, and positive regulation of NF-kappaB transcription factor activity (50). It is a major membrane-intrinsic protein located in caveolae, which are invaginations on the cell surface responsible for maintaining their integrity, facilitating small molecule transport, and mediating signaling processes. Microcaveolae are specialized intracellular depressions found on the surface of lipid rafts in the cell membrane (51). CAV1, an integral membrane protein primarily located on the cytosolic membrane, is a key component of microcyst formation and serves as one of the primary regulators of cellular senescence (52). Previous research indicates that CAV1 can modulate iron death by influencing reactive nitrogen species (RNS) metabolism during acute immune-mediated hepatic damage and liver fibrosis (25, 53). Given the highly metabolic nature of organs, particularly transplanted kidneys characterized by complex tissue infiltration of inflammatory factors, ferroptosis and other forms of cell death exhibit intricate interactions. Currently, some iron chelators and redox agents are mainly used to regulate the occurrence of ferroptosis (54), but specific targeted drugs have not yet been applied to the clinic. CAV1 emerges as a potential target for regulating cellular metabolism in the treatment of kidney diseases (55). Future studies should explore pharmacological inhibition of CAV1 or ferroptosis modulators to mitigate CAD progression.

In this study, we identified differentially expressed ferritin-related genes in CAD using publicly available data from the GEO database. The prediction model constructed based on these genes demonstrated good diagnostic efficacy for CAD and significant discriminatory power for the survival time of transplanted kidneys. Subsequently, we investigated the role of CAV1 in influencing the process of epithelial cell iron death in transplanted kidneys using clinical samples, animal models, and *in vitro* cellular experiments. Our findings elucidated the occurrence of iron death in renal tubular epithelial cells through the secretion of pro-fibrotic factors, resulting in epithelial-mesenchymal transition (EMT) in neighboring cells and accelerating the fibrosis of transplanted kidneys. However, this study has certain limitations, such as the intrinsic mechanism by which CAV1 regulates ferroptosis, warranting further investigation in subsequent studies, Moreover, this experiment is a single-center data study, needs to be validated in larger, more ethnically diverse groups.

## 5 Conclusions

In this study, we identified ferroptosis-related differential genes through analysis of the gene expression matrix of transplanted kidney puncture biopsy samples from the GEO database. We established a prognostic model for chronic allograft dysfunction (CAD) of the transplanted kidney by analyzing dataset GSE21374 and conducted survival analysis based on the expression levels of target genes. Subsequently, we selected CAV1 as a focus for further investigation and validated its effects using clinical samples, animal models, and cellular experiments. The findings suggest that elevated expression of CAV1 promotes epithelial-mesenchymal transition (EMT) in epithelial cells by activating the ferroptosis pathway, thereby contributing to the progression of CAD in transplanted kidneys.

## Data Availability

The original contributions presented in the study are included in the article/**Supplementary Materials**. Further inquiries can be directed to the corresponding authors.

## Supplementary material

Supplementary Figure 1 Mechanism diagram of CAV1 promotes epithelial-to-mesenchymal transition (EMT) and chronic renal allograft interstitial fibrosis by activating the ferroptosis pathway.

Supplementary Figure 2 Mouse animal model and knockdown and overexpression cell lines of HK-2 cells **(A, B)** Images of renal artery before and after opening blood flow in a mouse animal model. **(C, D)** Optical microscopy images and EGFP of control groups(NC) and CAV1 knockdown groups(Ri-CAV1) in HK-2 cells. **(E, F)** Optical microscopy images and EGFP of control groups(NC) and CAV1 overexpression groups(OE-CAV1) in HK-2 cells.

Supplementary Figure 3 Densitometric analysis for western blots in **Figure 7** **(A-C)** Protein levels of Fibronectin, E-cadherin and α-SMA in cell culture from the control groups(NC) and CAV1 knockdown groups(Ri-CAV1) treated with Erastin (5 μM) were analyzed by western blot assay. **(D-F)** Protein levels of Fibronectin, E-cadherin and α-SMA in cell culture from the control groups(NC) and CAV1 overexpression groups(OE-CAV1)), treated with Ferrostatin-1 (2 μM) were analyzed by western blot assay. **(G-I)** Protein levels of Fibronectin, E-cadherin and α-SMA in HK-2 cells from the co-culture (control groups(NC) and CAV1 knockdown groups(Ri-CAV1) treated with Erastin (5 μM))**. (J-L)** Protein levels of Fibronectin, E-cadherin and α-SMA in HK-2 cells from the co-culture (control groups(NC) and CAV1 overexpression groups(OE-CAV1)), treated with Ferrostatin-1 (2 μM)). **(M-O)** Protein levels of Fibronectin, E-cadherin and α-SMA in HK-2 cells from the co-culture (control groups(NC) and CAV1 overexpression groups(OE-CAV1)), treated with neutralizing antibodies of IL-6).

Supplementary Figure 4 Consolidated heatmap showing CAV1 expression across clinical and experimental groups. **(A)** Heatmap of CAV1 expression in GSE21374. **(B)** Heatmap of CAV1 expression in clinical samples. **(C)** Heatmap of CAV1 expression in animal models.

Supplementary Figure 5 Ferroptosis inhibitors could attenuates the occurrence of ferroptosis and the progression of EMT in mouse kidney transplant tissues **(A)** Representative immunofluorescence images of GPX4(green) expression in renal tissue from Syn,Allo, Syn +Fer-1 and Allo +Fer-1 groups; **(B)** The MDA levels were detected in renal tissues from the n Syn,Allo, Syn +Fer-1 and Allo +Fer-1 groups. **(C)** Levels of IL-6 in mouse serum were analyzed by ELISA assay. (D-E) Protein levels of E-cadherin and α-SMA in renal tissue from Syn,Allo, Syn +Fer-1 and Allo +Fer-1 groups, ns (no significance),*P < 0.05, ** P < 0.01, *** P < 0.001.

Supplementary Figure 6 The viability of each group of cells and performed quantitative analysis. **(A)** Representative Calcein AM (Calcein) and propidium iodide (PI) dual fluorescence of control groups(NC) and CAV1 knockdown groups(Ri-CAV1) treated with Erastin (5μM), control groups(NC) and CAV1 overexpression groups(OE-CAV1)), treated with Ferrostatin-1 (2 μM); Live cells show green fluorescence, dead cells show red fluorescence **(B)** Quantitative analysis of cell viability, ns (no significance),*P < 0.05, ** P < 0.01, *** P < 0.001.
