# Supplementary figures and images for: CAV1 promotes epithelial-to-mesenchymal transition (EMT) and chronic renal allograft interstitial fibrosis by activating the ferroptosis pathway
Source: Front Immunol. 2025 Feb 12;16:1523855. doi: 10.3389/fimmu.2025.1523855 (PMC11860899; doi:10.3389/fimmu.2025.1523855)

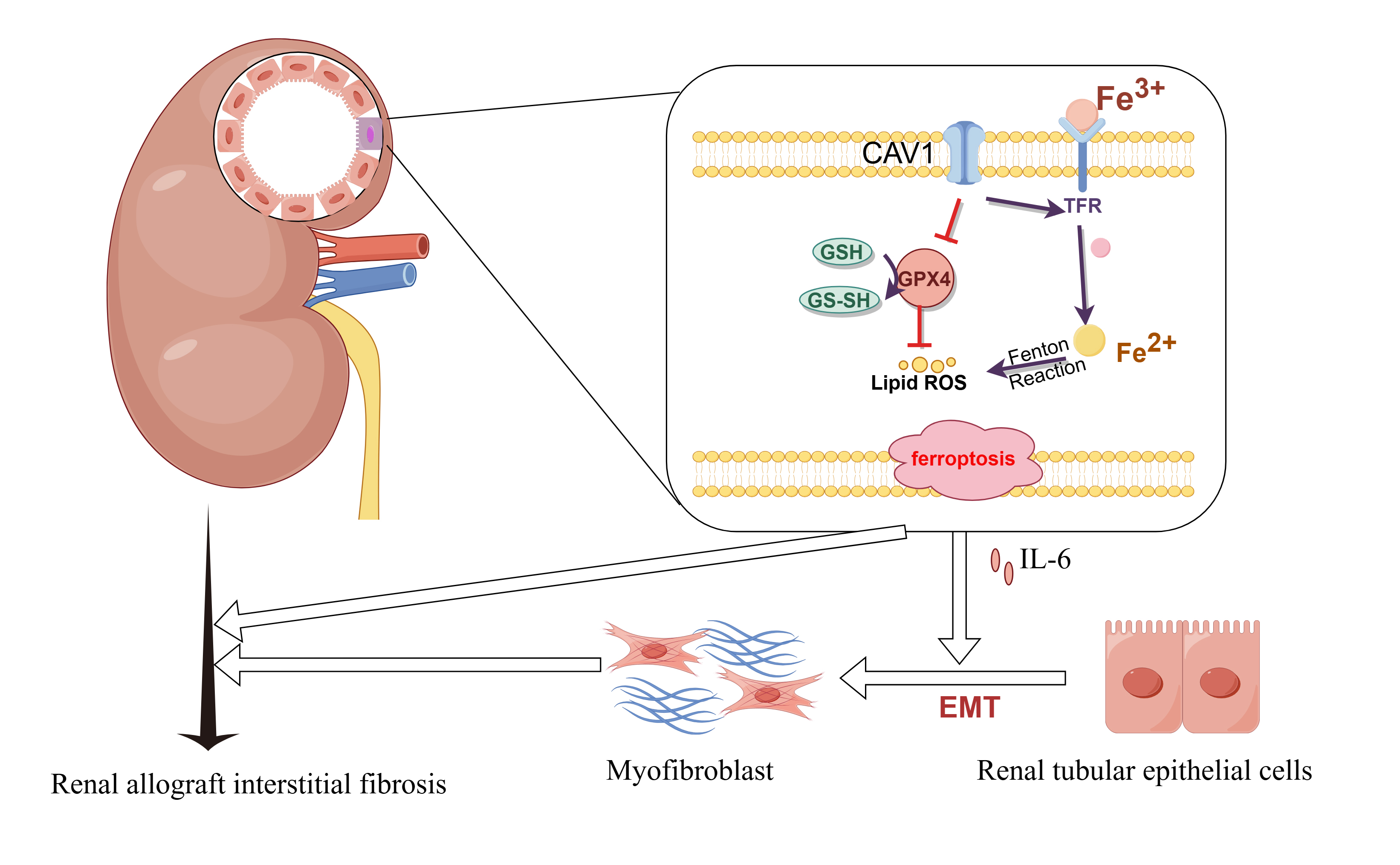

Supplement: Supplementary Figure 1 — Mechanism diagram of CAV1 promotes epithelial-to-mesenchymal transition (EMT) and chronic renal allograft interstitial fibrosis by activating the ferroptosis pathway. [file Image1.jpeg]

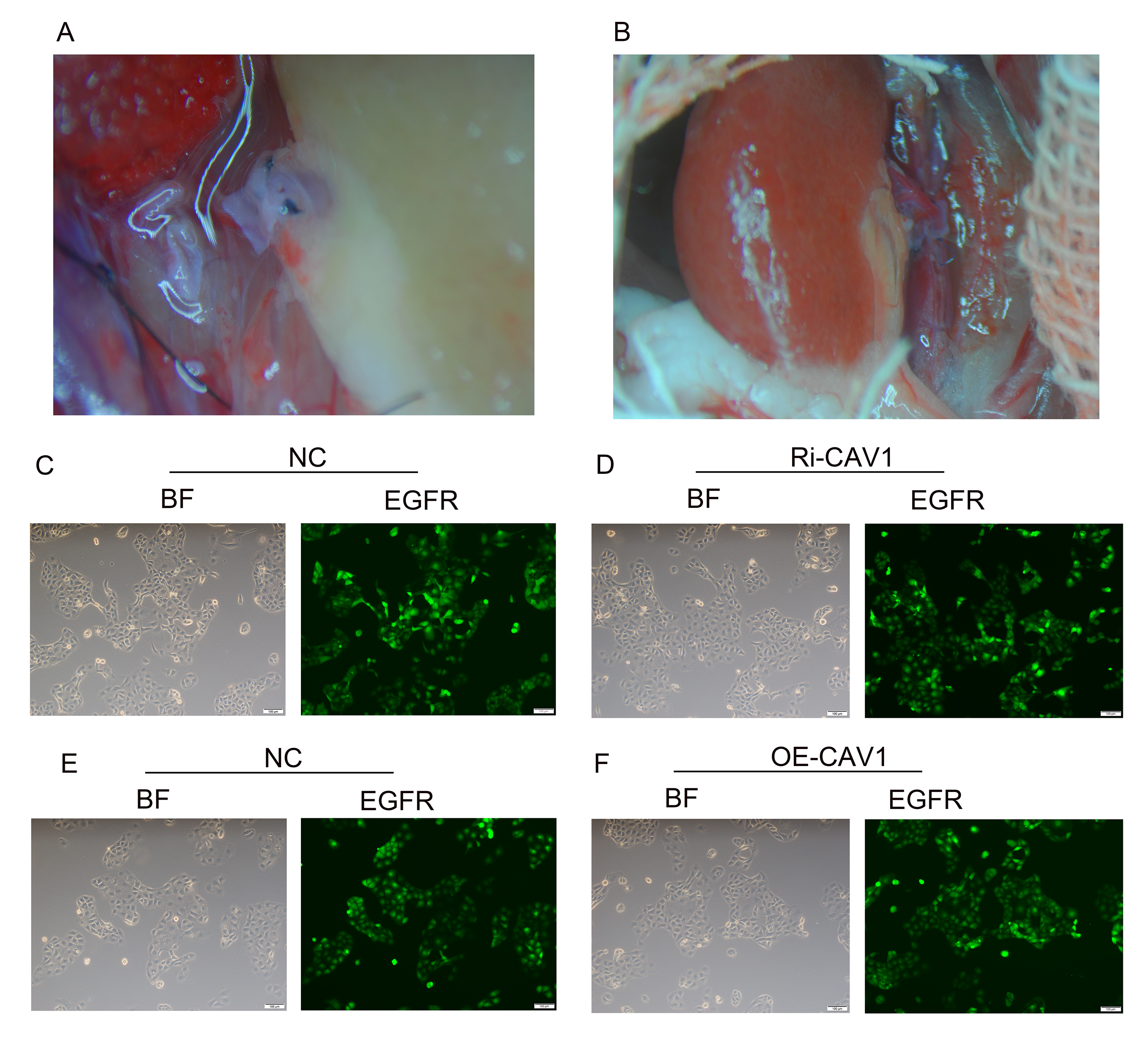

Supplement: Supplementary Figure 2 — Mouse animal model and knockdown and overexpression cell lines of HK-2 cells (A, B) Images of renal artery before and after opening blood flow in a mouse animal model. (C, D) Optical microscopy images and EGFP of control groups(NC) and CAV1 knockdown groups(Ri-CAV1) in HK-2 cells. (E, F) Optical microscopy images and EGFP of control groups(NC) and CAV1 overexpression groups(OE-CAV1) in HK-2 cells. [file Image2.jpeg]

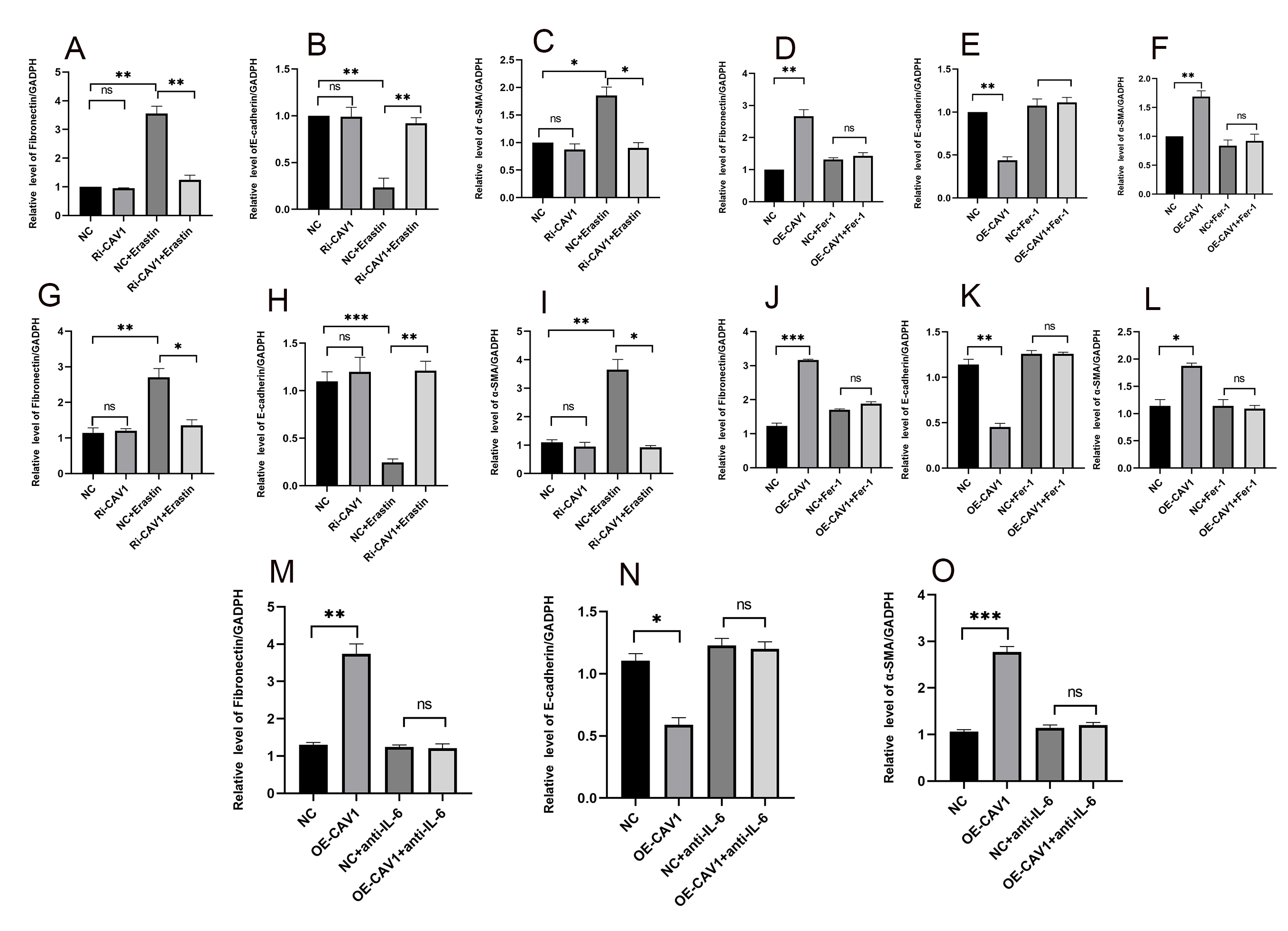

Supplement: Supplementary Figure 3 — Densitometric analysis for western blots in Figure 7 (A-C) Protein levels of Fibronectin, E-cadherin and α-SMA in cell culture from the control groups(NC) and CAV1 knockdown groups(Ri-CAV1) treated with Erastin (5 μM) were analyzed by western blot assay. (D-F) Protein levels of Fibronectin, E-cadherin and α-SMA in cell culture from the control groups(NC) and CAV1 overexpression groups(OE-CAV1)), treated with Ferrostatin-1 (2 μM) were analyzed by western blot assay. (G-I) Protein levels of Fibronectin, E-cadherin and α-SMA in HK-2 cells from the co-culture (control groups(NC) and CAV1 knockdown groups(Ri-CAV1) treated with Erastin (5 μM)). (J-L) Protein levels of Fibronectin, E-cadherin and α-SMA in HK-2 cells from the co-culture (control groups(NC) and CAV1 overexpression groups(OE-CAV1)), treated with Ferrostatin-1 (2 μM)). (M-O) Protein levels of Fibronectin, E-cadherin and α-SMA in HK-2 cells from the co-culture (control groups(NC) and CAV1 overexpression groups(OE-CAV1)), treated with neutralizing antibodies of IL-6). [file Image3.jpeg]

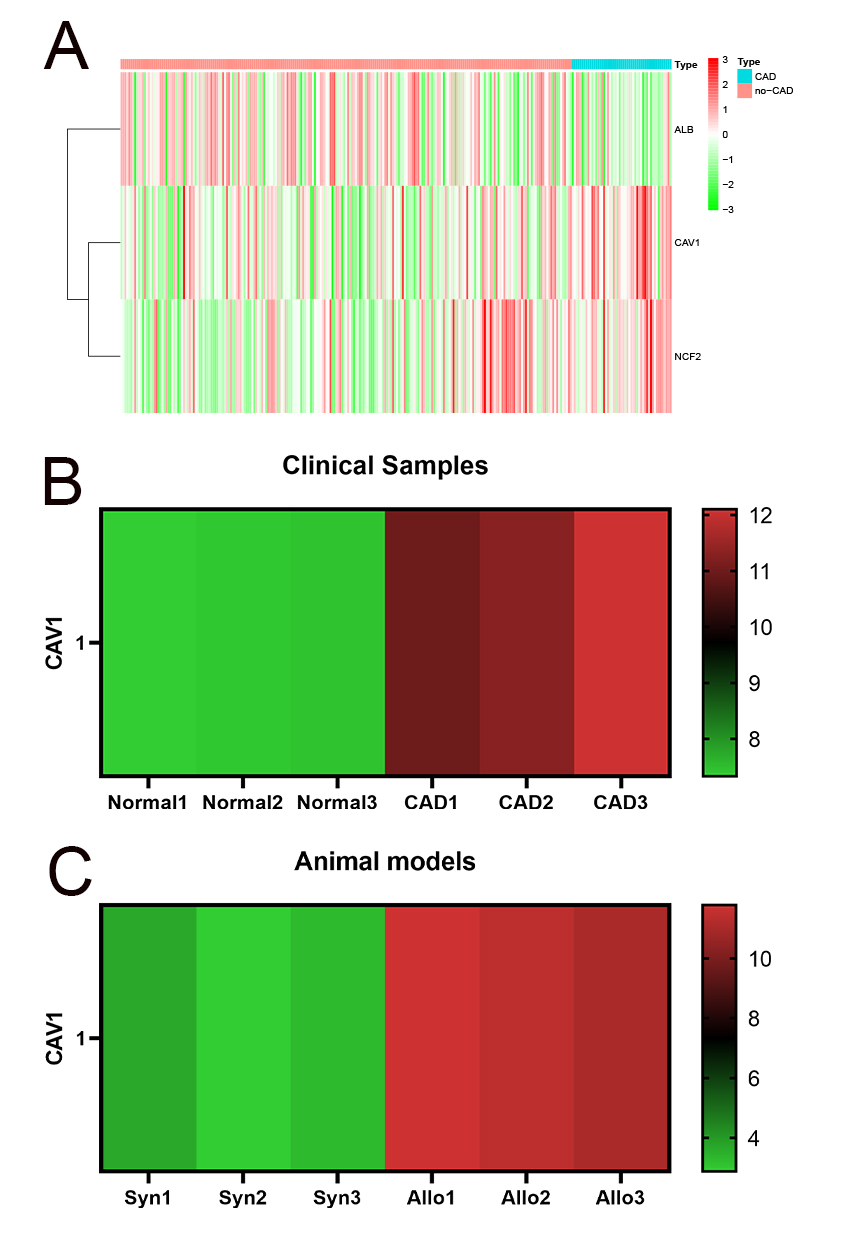

Supplement: Supplementary Figure 4 — Consolidated heatmap showing CAV1 expression across clinical and experimental groups. (A) Heatmap of CAV1 expression in GSE21374. (B) Heatmap of CAV1 expression in clinical samples. (C) Heatmap of CAV1 expression in animal models. [file Image4.jpeg]

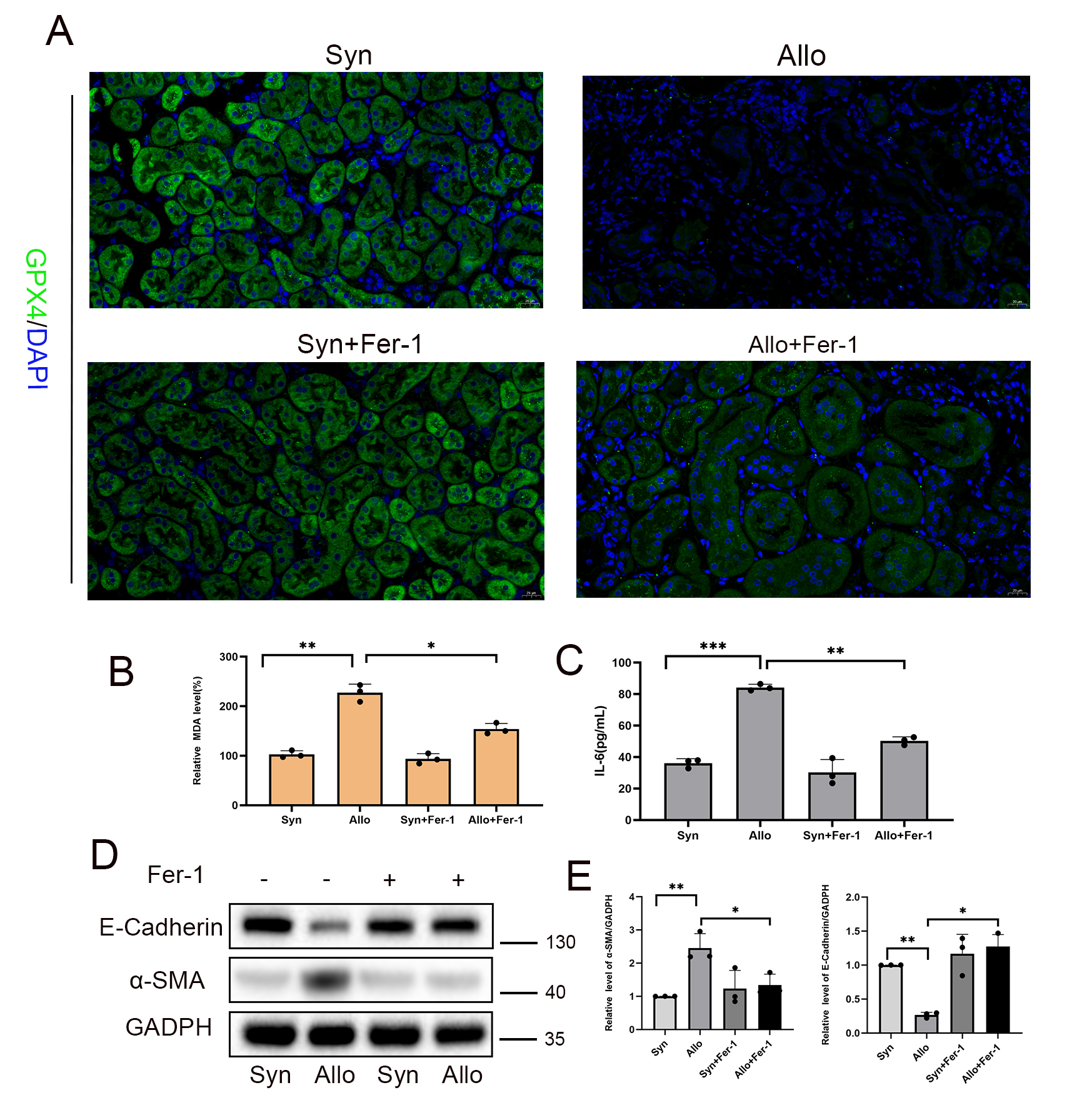

Supplement: Supplementary Figure 5 — Ferroptosis inhibitors could attenuates the occurrence of ferroptosis and the progression of EMT in mouse kidney transplant tissues (A) Representative immunofluorescence images of GPX4(green) expression in renal tissue from Syn,Allo, Syn +Fer-1 and Allo +Fer-1 groups; (B) The MDA levels were detected in renal tissues from the n Syn,Allo, Syn +Fer-1 and Allo +Fer-1 groups. (C) Levels of IL-6 in mouse serum were analyzed by ELISA assay. (D-E) Protein levels of E-cadherin and α-SMA in renal tissue from Syn,Allo, Syn +Fer-1 and Allo +Fer-1 groups, ns (no significance),*P < 0.05, ** P < 0.01, *** P < 0.001. [file Image5.jpeg]

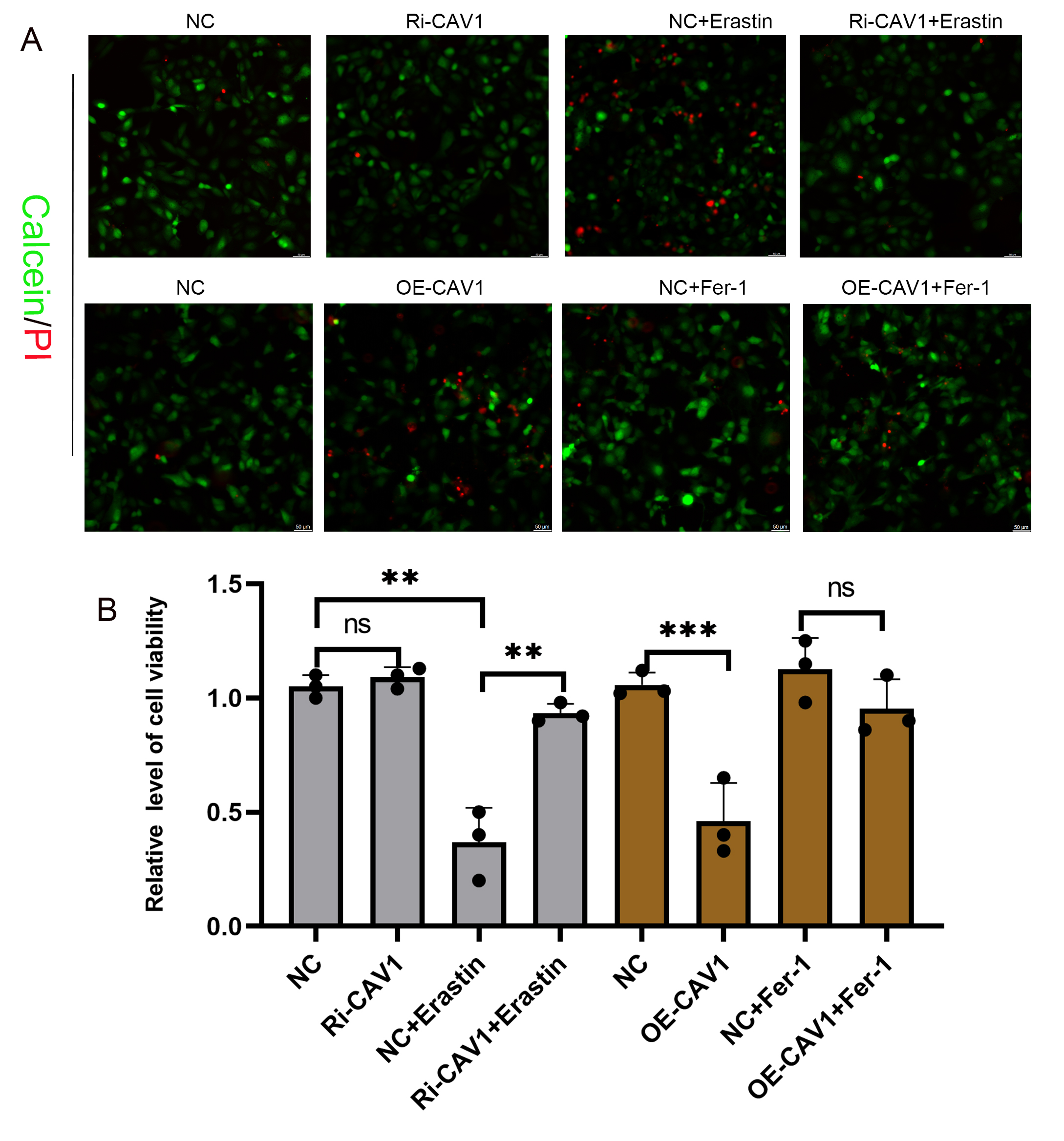

Supplement: Supplementary Figure 6 — The viability of each group of cells and performed quantitative analysis. (A) Representative Calcein AM (Calcein) and propidium iodide (PI) dual fluorescence of control groups(NC) and CAV1 knockdown groups(Ri-CAV1) treated with Erastin (5μM), control groups(NC) and CAV1 overexpression groups(OE-CAV1)), treated with Ferrostatin-1 (2 μM); Live cells show green fluorescence, dead cells show red fluorescence (B) Quantitative analysis of cell viability, ns (no significance),*P < 0.05, ** P < 0.01, *** P < 0.001. [file Image6.jpeg]
