# Supplementary material for: CAV1 promotes epithelial-to-mesenchymal transition (EMT) and chronic renal allograft interstitial fibrosis by activating the ferroptosis pathway
Source: Front Immunol. 2025 Feb 12;16:1523855. doi: 10.3389/fimmu.2025.1523855 (PMC11860899; doi:10.3389/fimmu.2025.1523855)
Supplement: Supplementary file 7 [file Table1.docx]

| **Supplementary file 1: Primer sequences** | |
| --- | --- |
| CAV1-Forward | GCGACCCTAAACACCTCAAC |
| CAV1-Reverse | ATGCCGTCAAAACTGTGTGTC |
| ALB-Forward | GAGACCAGAGGTTGATGTGATG |
| ALB-Reverse | AGTTCCGGGGCATAAAAGTAAG |
| NCF2-Forward | CCCACTCCCGGATTTGCTTC |
| NCF2-Reverse | GTCTCGGTTAATGCTTCTGGTAA |
