# Supplementary material for: CAV1 promotes epithelial-to-mesenchymal transition (EMT) and chronic renal allograft interstitial fibrosis by activating the ferroptosis pathway
Source: Front Immunol. 2025 Feb 12;16:1523855. doi: 10.3389/fimmu.2025.1523855 (PMC11860899; doi:10.3389/fimmu.2025.1523855)
Supplement: Supplementary file 8 [file Table2.docx]

**Supplementary file 2**

| **Antibodies information and dilution ratios** | | | |
| --- | --- | --- | --- |
| Antibody Name | CAT | Mfrs | Dilution(Western blot) |
| Caveolin-1 | 16447-1-AP | Proteintech | 1:1000 |
| Glutathione Peroxidase 4 (GPX4) | ab125066 | Abcam | 1:1000 |
| transferrin receptor (TFR) | 66180-1-Ig | Proteintech | 1:5000 |
| α-SMA | 40482 | SAB | 1:4000 |
| Fibronectin | 15613-1-AP | Proteintech | 1:2000 |
| E-cadherin | 40860 | SAB | 1:1000 |
| **Cell Lines** | | | |
| HK-2 cells (human proximal tubule epithelial cells) | Cell Bank of the Chinese Academy of Sciences | | Cat. SCSP-511 |
| **Key Reagents** | | | |
| Reagents | CAT | | Mfrs |
| fetal bovine serum (FBS) | FBS500-S | | AusGeneX |
| penicillin-streptomycin | BL505A | | Biosharp |
| DMEM/F12 | BL305A | | Biosharp |
| Ferrostatin-1 | HY-100579 | | MedChemExpress |
| Erastin | HY-15763 | | MedChemExpress |
| PVDF membrane | GVWP04700 | | Millipore |
| Exposure solution | FD8020 | | Fdbio |
| IHC secondary antibody Kit | abs996 | | Absin |
| diaminobenzidine (DAB) | abs9210 | | Absin |
| DAPI | C1002 | | Beyotime |
