# Supplementary material for: CAV1 promotes epithelial-to-mesenchymal transition (EMT) and chronic renal allograft interstitial fibrosis by activating the ferroptosis pathway
Source: Front Immunol. 2025 Feb 12;16:1523855. doi: 10.3389/fimmu.2025.1523855 (PMC11860899; doi:10.3389/fimmu.2025.1523855)
Supplement: Supplementary file 9 [file Table3.docx]

**Supplementary file 3**

**Univariate Cox regression results**

| id | HR | HR.95L | HR.95H | pvalue |
| --- | --- | --- | --- | --- |
| CAV1 | 2.043709 | 1.538383 | 2.715024 | 8.13E-07 |
| TNFAIP3 | 2.612071 | 1.851589 | 3.684897 | 4.53E-08 |
| ALB | 0.633436 | 0.515566 | 0.778253 | 1.38E-05 |
| PCK2 | 0.590961 | 0.479519 | 0.728303 | 8.07E-07 |
| NCF2 | 2.283122 | 1.720588 | 3.029572 | 1.07E-08 |
| MIOX | 0.711724 | 0.605918 | 0.836007 | 3.45E-05 |

**Multivariate Cox regression results**

| id | coef | HR | HR.95L | HR.95H | pvalue |
| --- | --- | --- | --- | --- | --- |
| CAV1 | 0.39235 | 1.480456 | 1.063562 | 2.060763 | 0.020063 |
| ALB | -0.22547 | 0.798141 | 0.633807 | 1.005083 | 0.055256 |
| NCF2 | 0.582425 | 1.790376 | 1.293818 | 2.477509 | 0.000441 |
